# 1-(1,3-Benzothia­zol-2-yl)-3-phenyl-2-pyrazoline

**DOI:** 10.1107/S1600536812013219

**Published:** 2012-03-31

**Authors:** Dong-Feng Li, Xiao-Fei Zhu, Shuang Guan, Rui-Bin Hou

**Affiliations:** a School of Chemistry and Life Science, Changchun University of Technology, Changchun 130012, People’s Republic of China

## Abstract

In the title compound, C_16_H_13_N_3_S, the pyrazoline ring forms dihedral angles of 6.89 (14) and 4.96 (11)° with the benzene ring and the benzothia­zole group, respectively. In the crystal, weak C—H⋯N inter­actions link the mol­ecules into chains extending along the *b*-axis direction.

## Related literature

For background to the title compound, see: Sano *et al.* (1995 ▶); Xian *et al.* (2008 ▶). For details of the synthesis, see: Xian *et al.* (2008 ▶).
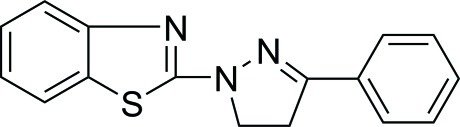


## Experimental

### 

#### Crystal data

C_16_H_13_N_3_S
*M* *_r_* = 279.35
Monoclinic, 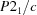

*a* = 16.946 (8) Å
*b* = 5.449 (3) Å
*c* = 17.306 (11) Å
β = 119.96 (2)°
*V* = 1384.4 (14) Å^3^
*Z* = 4
Mo *K*α radiation
μ = 0.23 mm^−1^
*T* = 288 K
0.54 × 0.30 × 0.28 mm

#### Data collection

Rigaku R-AXIS RAPID diffractometer
Absorption correction: multi-scan (*ABSCOR*; Higashi, 1995 ▶) *T*_min_ = 0.887, *T*_max_ = 0.938
11972 measured reflections
3141 independent reflections
2227 reflections with *I* > 2σ(*I*)
*R*_int_ = 0.028

#### Refinement

*R*[*F* ^2^ > 2σ(*F* ^2^)] = 0.039
*wR*(*F* ^2^) = 0.116
*S* = 1.14
3141 reflections
181 parameters
H-atom parameters constrained
Δρ_max_ = 0.20 e Å^−3^
Δρ_min_ = −0.20 e Å^−3^

### 

Data collection: *RAPID-AUTO* (Rigaku, 1998 ▶); cell refinement: *RAPID-AUTO*; data reduction: *CrystalStructure* (MSC & Rigaku, 2002 ▶); program(s) used to solve structure: *SHELXS97* (Sheldrick, 2008 ▶); program(s) used to refine structure: *SHELXL97* (Sheldrick, 2008 ▶); molecular graphics: *SHELXTL* (Sheldrick, 2008 ▶); software used to prepare material for publication: *SHELXL97*.

## Supplementary Material

Crystal structure: contains datablock(s) global, I. DOI: 10.1107/S1600536812013219/gk2466sup1.cif

Structure factors: contains datablock(s) I. DOI: 10.1107/S1600536812013219/gk2466Isup2.hkl

Supplementary material file. DOI: 10.1107/S1600536812013219/gk2466Isup3.cml

Additional supplementary materials:  crystallographic information; 3D view; checkCIF report

## Figures and Tables

**Table 1:** Hydrogen-bond geometry (Å, °)

*D*—H⋯*A*	*D*—H	H⋯*A*	*D*⋯*A*	*D*—H⋯*A*
C2—H2⋯N3^i^	0.93	2.61	3.340 (3)	135

Symmetry code: (i) 
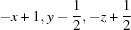
.

**Fig. 1.**
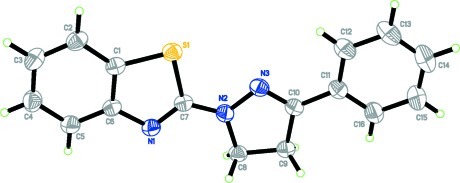
Molecular structure of the title compound with displacement ellipsoids drawn at the 30% probalility level.

## supplementary crystallographic information

### Comment

Pyrazoline derivatives have been investigated in many repects due to their blue light emission with high quantum yield, accessibility. They are used as carrier transporting as well as emitting materials (Sano *et al.*, 1995). Recently, Xian reported the synthesis and optical properties of novel pyrazoline derivatives as blue light fluorescence compounds (Xian *et al.*, 2008). In this paper, we describe the crystal structure of the title compound with blue light fluorescence.

The molecular structure of title compound, C_16_H_13_N_3_S, is shown in Fig. 1, all bond lengths and angles are in the normal ranges. The pyrazoline ring and benzothiazole ring are nearly coplanar and make dihedral angle of 4.96 (11)°. The molecules are linked by intermolecular C—H···N hydrogen bonds (Table 1), generating chains along the *b* direction.

### Experimental

The title compound was prepared according to the literature (Xian *et al.*, 2008). Single crystals suitable for X-ray diffraction were prepared by slow evaporation method from a solution in dichloromethane/petroleum (60–90 °C) at room temperature.

### Refinement

Carbon-bound H-atoms were placed in calculated positions (C—H 0.93 and 0.97 Å) and were included in the refinement in the riding model approximation with *U*_iso_(H) = 1.2 *U*_eq_(C).

### Crystal data

**Table:**

C_16_H_13_N_3_S	*F*(000) = 584
*M**_r_* = 279.35	*D*_x_ = 1.340 Mg m^−^^3^
Monoclinic, *P*2_1_/*c*	Mo *K*α radiation, λ = 0.71073 Å
Hall symbol: -P 2ybc	Cell parameters from 8759 reflections
*a* = 16.946 (8) Å	θ = 3.6–27.6°
*b* = 5.449 (3) Å	µ = 0.23 mm^−^^1^
*c* = 17.306 (11) Å	*T* = 288 K
β = 119.96 (2)°	Block, colorless
*V* = 1384.4 (14) Å^3^	0.54 × 0.30 × 0.28 mm
*Z* = 4	

### Data collection

**Table:**

Rigaku R-AXIS RAPID diffractometer	3141 independent reflections
Radiation source: fine-focus sealed tube	2227 reflections with *I* > 2σ(*I*)
Graphite monochromator	*R*_int_ = 0.028
ω scans	θ_max_ = 27.5°, θ_min_ = 3.6°
Absorption correction: multi-scan (*ABSCOR*; Higashi, 1995)	*h* = −21→21
*T*_min_ = 0.887, *T*_max_ = 0.938	*k* = −7→6
11972 measured reflections	*l* = −22→22

### Refinement

**Table:**

Refinement on *F*^2^	Primary atom site location: structure-invariant direct methods
Least-squares matrix: full	Secondary atom site location: difference Fourier map
*R*[*F*^2^ > 2σ(*F*^2^)] = 0.039	Hydrogen site location: inferred from neighbouring sites
*wR*(*F*^2^) = 0.116	H-atom parameters constrained
*S* = 1.14	*w* = 1/[σ^2^(*F*_o_^2^) + (0.0607*P*)^2^ + 0.0076*P*] where *P* = (*F*_o_^2^ + 2*F*_c_^2^)/3
3141 reflections	(Δ/σ)_max_ < 0.001
181 parameters	Δρ_max_ = 0.20 e Å^−^^3^
0 restraints	Δρ_min_ = −0.20 e Å^−^^3^

### Special details

**Table:**

Experimental. (See detailed section in the paper)
Geometry. All e.s.d.'s (except the e.s.d. in the dihedral angle between two l.s. planes) are estimated using the full covariance matrix. The cell e.s.d.'s are taken into account individually in the estimation of e.s.d.'s in distances, angles and torsion angles; correlations between e.s.d.'s in cell parameters are only used when they are defined by crystal symmetry. An approximate (isotropic) treatment of cell e.s.d.'s is used for estimating e.s.d.'s involving l.s. planes.
Refinement. Refinement of *F*^2^ against ALL reflections. The weighted *R*-factor *wR* and goodness of fit *S* are based on *F*^2^, conventional *R*-factors *R* are based on *F*, with *F* set to zero for negative *F*^2^. The threshold expression of *F*^2^ > σ(*F*^2^) is used only for calculating *R*-factors(gt) *etc*. and is not relevant to the choice of reflections for refinement. *R*-factors based on *F*^2^ are statistically about twice as large as those based on *F*, and *R*- factors based on ALL data will be even larger.

### Fractional atomic coordinates and isotropic or equivalent isotropic displacement parameters (Å^2^)

**Table:**

	*x*	*y*	*z*	*U*_iso_*/*U*_eq_	
S1	0.50225 (3)	−0.17993 (8)	0.32651 (3)	0.05497 (16)	
N1	0.43537 (9)	0.1902 (2)	0.37317 (10)	0.0552 (4)	
N2	0.59086 (9)	0.1280 (3)	0.46547 (10)	0.0569 (4)	
N3	0.66870 (9)	0.0055 (2)	0.48121 (9)	0.0541 (3)	
C1	0.38592 (10)	−0.1199 (3)	0.26333 (11)	0.0502 (4)	
C2	0.32007 (12)	−0.2465 (4)	0.18951 (13)	0.0617 (5)	
H2	0.3354	−0.3833	0.1677	0.074*	
C3	0.23125 (12)	−0.1641 (4)	0.14930 (13)	0.0678 (5)	
H3	0.1862	−0.2452	0.0994	0.081*	
C4	0.20868 (12)	0.0380 (4)	0.18261 (13)	0.0690 (5)	
H4	0.1485	0.0913	0.1544	0.083*	
C5	0.27335 (12)	0.1621 (3)	0.25656 (13)	0.0629 (5)	
H5	0.2570	0.2966	0.2786	0.075*	
C6	0.36345 (10)	0.0836 (3)	0.29788 (11)	0.0509 (4)	
C7	0.50936 (10)	0.0702 (3)	0.39398 (11)	0.0499 (4)	
C8	0.60831 (11)	0.3438 (3)	0.52172 (12)	0.0554 (4)	
H8A	0.5770	0.3329	0.5557	0.066*	
H8B	0.5901	0.4934	0.4867	0.066*	
C9	0.71109 (11)	0.3312 (3)	0.58214 (12)	0.0568 (4)	
H9A	0.7402	0.4805	0.5784	0.068*	
H9B	0.7274	0.3038	0.6437	0.068*	
C10	0.73713 (11)	0.1147 (3)	0.54526 (11)	0.0493 (4)	
C11	0.83120 (11)	0.0387 (3)	0.57541 (12)	0.0546 (4)	
C12	0.85070 (13)	−0.1548 (4)	0.53559 (14)	0.0704 (5)	
H12	0.8037	−0.2453	0.4906	0.085*	
C13	0.94034 (15)	−0.2128 (5)	0.56296 (17)	0.0892 (7)	
H13	0.9533	−0.3426	0.5362	0.107*	
C14	1.01079 (15)	−0.0798 (5)	0.62958 (18)	0.0935 (7)	
H14	1.0709	−0.1183	0.6471	0.112*	
C15	0.99182 (13)	0.1075 (5)	0.66937 (17)	0.0882 (7)	
H15	1.0392	0.1961	0.7147	0.106*	
C16	0.90289 (12)	0.1677 (4)	0.64329 (14)	0.0725 (5)	
H16	0.8909	0.2958	0.6714	0.087*	

### Atomic displacement parameters (Å^2^)

**Table:**

	*U*^11^	*U*^22^	*U*^33^	*U*^12^	*U*^13^	*U*^23^
S1	0.0594 (3)	0.0536 (3)	0.0590 (3)	0.00083 (18)	0.0349 (2)	−0.00678 (19)
N1	0.0564 (8)	0.0489 (8)	0.0602 (9)	−0.0009 (6)	0.0290 (7)	−0.0076 (6)
N2	0.0519 (7)	0.0566 (8)	0.0600 (9)	0.0041 (6)	0.0262 (7)	−0.0112 (7)
N3	0.0600 (8)	0.0525 (8)	0.0543 (8)	0.0057 (6)	0.0318 (7)	−0.0010 (6)
C1	0.0578 (9)	0.0483 (9)	0.0526 (9)	−0.0047 (7)	0.0336 (8)	−0.0014 (7)
C2	0.0682 (11)	0.0625 (10)	0.0650 (12)	−0.0099 (8)	0.0413 (9)	−0.0154 (9)
C3	0.0627 (10)	0.0779 (13)	0.0625 (12)	−0.0164 (9)	0.0311 (9)	−0.0172 (9)
C4	0.0552 (10)	0.0773 (13)	0.0703 (13)	−0.0026 (9)	0.0281 (9)	−0.0058 (10)
C5	0.0601 (9)	0.0554 (11)	0.0721 (12)	0.0015 (8)	0.0322 (9)	−0.0074 (9)
C6	0.0569 (9)	0.0448 (9)	0.0544 (10)	−0.0052 (7)	0.0304 (8)	−0.0018 (7)
C7	0.0570 (9)	0.0450 (9)	0.0533 (10)	−0.0026 (7)	0.0317 (8)	−0.0020 (7)
C8	0.0613 (9)	0.0485 (9)	0.0548 (10)	0.0041 (7)	0.0279 (8)	−0.0052 (7)
C9	0.0593 (9)	0.0516 (10)	0.0588 (11)	0.0004 (7)	0.0291 (8)	−0.0049 (8)
C10	0.0564 (9)	0.0482 (9)	0.0478 (9)	0.0045 (7)	0.0294 (7)	0.0060 (7)
C11	0.0592 (9)	0.0552 (10)	0.0550 (10)	0.0082 (8)	0.0327 (8)	0.0117 (8)
C12	0.0716 (11)	0.0737 (13)	0.0710 (13)	0.0159 (9)	0.0393 (10)	0.0026 (10)
C13	0.0864 (15)	0.0968 (17)	0.0963 (18)	0.0324 (13)	0.0546 (14)	0.0062 (14)
C14	0.0644 (12)	0.118 (2)	0.1009 (19)	0.0243 (13)	0.0430 (13)	0.0210 (16)
C15	0.0573 (11)	0.0989 (16)	0.0930 (17)	0.0079 (11)	0.0259 (11)	0.0044 (14)
C16	0.0586 (10)	0.0765 (13)	0.0736 (14)	0.0073 (9)	0.0264 (10)	0.0004 (10)

### Geometric parameters (Å, º)

**Table:**

S1—C1	1.7424 (18)	C8—C9	1.521 (2)
S1—C7	1.7591 (18)	C8—H8A	0.9700
N1—C7	1.296 (2)	C8—H8B	0.9700
N1—C6	1.391 (2)	C9—C10	1.508 (2)
N2—C7	1.353 (2)	C9—H9A	0.9700
N2—N3	1.3788 (18)	C9—H9B	0.9700
N2—C8	1.459 (2)	C10—C11	1.468 (2)
N3—C10	1.282 (2)	C11—C12	1.387 (3)
C1—C2	1.389 (2)	C11—C16	1.387 (3)
C1—C6	1.400 (2)	C12—C13	1.383 (3)
C2—C3	1.380 (3)	C12—H12	0.9300
C2—H2	0.9300	C13—C14	1.380 (3)
C3—C4	1.382 (3)	C13—H13	0.9300
C3—H3	0.9300	C14—C15	1.356 (4)
C4—C5	1.377 (2)	C14—H14	0.9300
C4—H4	0.9300	C15—C16	1.379 (3)
C5—C6	1.391 (2)	C15—H15	0.9300
C5—H5	0.9300	C16—H16	0.9300
			
C1—S1—C7	87.26 (8)	N2—C8—H8B	111.4
C7—N1—C6	108.86 (14)	C9—C8—H8B	111.4
C7—N2—N3	120.46 (14)	H8A—C8—H8B	109.3
C7—N2—C8	124.87 (14)	C10—C9—C8	102.76 (13)
N3—N2—C8	113.78 (13)	C10—C9—H9A	111.2
C10—N3—N2	108.00 (14)	C8—C9—H9A	111.2
C2—C1—C6	121.40 (15)	C10—C9—H9B	111.2
C2—C1—S1	128.44 (14)	C8—C9—H9B	111.2
C6—C1—S1	110.15 (12)	H9A—C9—H9B	109.1
C3—C2—C1	118.38 (17)	N3—C10—C11	121.95 (15)
C3—C2—H2	120.8	N3—C10—C9	113.54 (13)
C1—C2—H2	120.8	C11—C10—C9	124.44 (15)
C2—C3—C4	120.52 (17)	C12—C11—C16	118.72 (16)
C2—C3—H3	119.7	C12—C11—C10	121.59 (17)
C4—C3—H3	119.7	C16—C11—C10	119.65 (16)
C5—C4—C3	121.41 (17)	C13—C12—C11	119.8 (2)
C5—C4—H4	119.3	C13—C12—H12	120.1
C3—C4—H4	119.3	C11—C12—H12	120.1
C4—C5—C6	119.12 (16)	C14—C13—C12	120.7 (2)
C4—C5—H5	120.4	C14—C13—H13	119.7
C6—C5—H5	120.4	C12—C13—H13	119.7
N1—C6—C5	125.20 (15)	C15—C14—C13	119.6 (2)
N1—C6—C1	115.65 (14)	C15—C14—H14	120.2
C5—C6—C1	119.14 (15)	C13—C14—H14	120.2
N1—C7—N2	122.74 (15)	C14—C15—C16	120.7 (2)
N1—C7—S1	118.07 (12)	C14—C15—H15	119.7
N2—C7—S1	119.18 (12)	C16—C15—H15	119.7
N2—C8—C9	101.73 (12)	C15—C16—C11	120.6 (2)
N2—C8—H8A	111.4	C15—C16—H16	119.7
C9—C8—H8A	111.4	C11—C16—H16	119.7
			
C7—N2—N3—C10	171.82 (15)	C8—N2—C7—S1	173.69 (13)
C8—N2—N3—C10	2.14 (19)	C1—S1—C7—N1	−0.77 (13)
C7—S1—C1—C2	−178.47 (17)	C1—S1—C7—N2	178.58 (14)
C7—S1—C1—C6	0.79 (12)	C7—N2—C8—C9	−173.15 (16)
C6—C1—C2—C3	1.2 (3)	N3—N2—C8—C9	−4.01 (18)
S1—C1—C2—C3	−179.59 (14)	N2—C8—C9—C10	3.99 (16)
C1—C2—C3—C4	−0.7 (3)	N2—N3—C10—C11	−176.45 (14)
C2—C3—C4—C5	−0.3 (3)	N2—N3—C10—C9	0.87 (19)
C3—C4—C5—C6	0.7 (3)	C8—C9—C10—N3	−3.27 (19)
C7—N1—C6—C5	179.52 (15)	C8—C9—C10—C11	173.97 (15)
C7—N1—C6—C1	0.2 (2)	N3—C10—C11—C12	0.4 (3)
C4—C5—C6—N1	−179.49 (17)	C9—C10—C11—C12	−176.66 (16)
C4—C5—C6—C1	−0.2 (3)	N3—C10—C11—C16	178.12 (17)
C2—C1—C6—N1	178.56 (15)	C9—C10—C11—C16	1.1 (2)
S1—C1—C6—N1	−0.76 (18)	C16—C11—C12—C13	−1.0 (3)
C2—C1—C6—C5	−0.8 (3)	C10—C11—C12—C13	176.74 (18)
S1—C1—C6—C5	179.88 (13)	C11—C12—C13—C14	0.0 (3)
C6—N1—C7—N2	−178.85 (15)	C12—C13—C14—C15	0.9 (4)
C6—N1—C7—S1	0.47 (18)	C13—C14—C15—C16	−0.6 (4)
N3—N2—C7—N1	−175.46 (14)	C14—C15—C16—C11	−0.4 (4)
C8—N2—C7—N1	−7.0 (3)	C12—C11—C16—C15	1.3 (3)
N3—N2—C7—S1	5.2 (2)	C10—C11—C16—C15	−176.54 (18)

### Hydrogen-bond geometry (Å, º)

**Table:**

*D*—H···*A*	*D*—H	H···*A*	*D*···*A*	*D*—H···*A*
C2—H2···N3^i^	0.93	2.61	3.340 (3)	135

Symmetry code: (i) −*x*+1, *y*−1/2, −*z*+1/2.
